# 543. Molnupiravir Maintains Antiviral Activity Against SARS-CoV-2 Variants In Vitro and in Early Clinical Studies

**DOI:** 10.1093/ofid/ofab466.742

**Published:** 2021-12-04

**Authors:** Jay Grobler, Julie Strizki, Nicholas Murgolo, Wei Gao, Youfang Cao, Ying Zhang, Jiejun Du, Manoj Nair, Yaoxing Huang, Yang Luo, Daria Hazuda, David D Ho, David D Ho

**Affiliations:** 1 Merck & Co., Inc., Kenilworth, New Jersey; 2 Merck & Co. Inc, West Point, Pennsylvania; 3 Aaron Diamond AIDS Research Center, Columbia University Vagelos College of Physicians and Surgeons, New York, New York; 4 Columbia University Vagelos College of Physicians and Surgeons , New York, NY

## Abstract

**Background:**

Molnupiravir (MOV, MK-4482, EIDD-2801) is an orally administered prodrug of N-hydroxycytidine (NHC, EIDD-1931), a nucleoside with broad antiviral activity against a range of RNA viruses. MOV acts by driving viral error catastrophe following its incorporation by the viral RdRp into the viral genome. Given its mechanism of action, MOV activity should not be affected by substitutions in the spike protein present in SARS-CoV-2 variants of concern which impact efficacy of therapeutic neutralizing antibodies and vaccine induced immunity. We characterized MOV activity against variants by assessing antiviral activity in vitro and virologic response from the Phase 2/3 clinical trials (MOVe-In, MOVe-Out) for treatment of COVID-19.

**Methods:**

MOV activity against several SARS-CoV-2 variants, was evaluated in an in vitro infection assay. Antiviral potency of NHC (IC50) was determined in Vero E6 cells infected with virus at MOI ~0.1 by monitoring CPE. Longitudinal SARS-CoV-2 RNA viral load measures in participants enrolled in MOVe-In and MOVe-Out were analyzed based on SARS-CoV-2 genotype. Sequences of SARS-CoV-2 from study participants were amplified from nasal swabs by PCR and NGS was performed on samples with viral genome RNA of >22,000 copies/ml amplified by primers covering full length genome with Ion Torrent sequencing to identify clades represented in trial participants. SARS-CoV-2 clades were assigned using clade.nextstrain.org.

**Results:**

In vitro, NHC was equally effective against SARS-CoV-2 variants B.1.1.7 (20I), B.1351 (20H), and P1 (20J), compared with the original WA1 (19B) isolate. In clinical trials, no discernable difference was observed in magnitude of viral response measured by change from baseline in RNA titer over time across all clades represented including 20A through 20E and 20G to 20I. No participants at the time of the study presented with 20F, 20J, or 21A.

**Conclusion:**

Distribution of clades in participants in MOVe-In and MOVe-Out was representative of those circulating globally at the time of collection (Oct 2020 – Jan 2021). Both in vitro and clinical data suggest that spike protein substitutions do not impact antiviral activity of MOV and suggest its potential use for the treatment of SARS-CoV-2 variants.

**Disclosures:**

**Jay Grobler, PhD**, **Merck & Co., Inc.** (Employee, Shareholder) **Julie Strizki, PhD**, **Merck & Co., Inc.** (Employee, Shareholder) **Nicholas Murgolo, PhD**, **Merck & Co., Inc.** (Employee, Shareholder) **Wei Gao, PhD**, **Merck & Co., Inc.** (Employee, Shareholder) **Youfang Cao, PhD**, **Merck & Co.** (Employee) **Ying Zhang, PhD**, **Merck & Co., Inc.** (Employee, Shareholder) **Jiejun Du, PhD**, **Merck & Co., Inc.** (Employee, Shareholder) **Manoj Nair, PhD**, **Merck & Co., Inc.** (Grant/Research Support, Scientific Research Study Investigator, Research Grant or Support) **Yaoxing Huang, PhD**, **Merck & Co., Inc.** (Grant/Research Support, Scientific Research Study Investigator, Research Grant or Support) **Yang Luo, PhD**, **Merck & Co., Inc.** (Grant/Research Support, Scientific Research Study Investigator, Research Grant or Support) **Daria Hazuda, PhD**, **Merck & Co., Inc.** (Employee, Shareholder) **David D. Ho, MD**, **Merck & Co., Inc.** (Grant/Research Support, Scientific Research Study Investigator, Research Grant or Support) **David D. Ho, MD**, Brii Biosciences (Individual(s) Involved: Self): Consultant; Merck (Individual(s) Involved: Self): Research Grant or Support; RenBio (Individual(s) Involved: Self): Consultant, Founder, Other Financial or Material Support, Shareholder; WuXi Biologics (Individual(s) Involved: Self): Consultant

